# Positive and Negative Affect Mediate the Influences of a Maladaptive Emotion Regulation Strategy on Sleep Quality

**DOI:** 10.3389/fpsyt.2019.00628

**Published:** 2019-08-30

**Authors:** Iqra Latif, Alun T. L. Hughes, Robert C. A. Bendall

**Affiliations:** ^1^Directorate of Psychology & Public Health, School of Health and Society, University of Salford, Salford, United Kingdom; ^2^School of Natural Sciences and Psychology, Faculty of Science, Liverpool John Moores University, Liverpool, United Kingdom

**Keywords:** sleep, expressive suppression, cognitive reappraisal, emotion, mental health, psychopathology, emotion regulation, mediation analysis

## Abstract

Positive affect, negative affect, and emotion regulation strategies are related to sleep quality. Emotion regulation can also act as either a protective factor against the development of psychopathologies, or as a risk factor for their development, and therefore may be one mechanism linking mental health and sleep. However, currently it is not known whether affect can mediate the impact of emotion regulation strategy use on sleep quality. An opportunity sample in a healthy population completed the Positive and Negative Affect Schedule providing measures of positive and negative affect, the Pittsburgh Sleep Quality Index providing a measure of sleep quality, and the Emotion Regulation Questionnaire to record habitual use of emotion regulation strategies. Data were analysed using regression and mediation analyses. Negative affect and expressive suppression were positively correlated with PSQI score suggesting that as negative affect and expressive suppression use increased, sleep quality decreased. Positive affect was negatively correlated with PSQI score suggesting that as positive affect increased sleep quality improved. Further, mediation analyses revealed that both positive affect and negative affect mediated the impact of expressive suppression on sleep quality. Moreover, this partial mediation provides the first description that the influences of affect and expressive suppression on sleep quality are at least partially distinct. Targeting improvements in negative affect and effective emotion regulation strategy use may improve the efficacy of interventions aimed at improving sleep quality and the reduction in symptomology in psychopathologies.

## Introduction

Sleep is essential for health and is related to cognitive performance, emotion regulation (ER) and quality of life (1). Different ER strategies, broadly defined as conscious or non-conscious efforts to start, stop, or modulate emotions (2), have been shown to protect individuals from developing psychopathologies including anxiety and depression, or act as a risk factor for their development (3). Poor sleep quality is linked to impaired emotional functioning and dysregulated ER which may lead to the development of depressive symptoms (4). Indeed, abnormalities in the function of brain structures and neurotransmitters involved in the sleep–wake cycle and affective disorders overlap [e.g. Refs. (5–8)].

The precise relationships between sleep quality and affect are currently not fully understood. Alvaro et al. (9) suggest that there is a bidirectional relationship between insomnia and affective disorders, suggesting that insomnia can predict prevalence of anxiety and depression and that in turn anxiety and depression can predict the prevalence of insomnia. Conversely, it has also been suggested that sleep quality can predict positive and negative affect but not vice versa (10). Interestingly, for individuals with insomnia and major depressive disorder, the treatment of insomnia with cognitive behavioral insomnia therapy has been shown to improve depressive symptoms even without simultaneous antidepressant pharmacotherapy (11). Additionally, impaired sleep quality has been shown to influence emotional reactivity to everyday events in healthy individuals and those with minor or major unipolar depression. Adopting an experience sampling method allowing emotional reactivity to daily events to be recorded throughout participants’ daily life, sleep difficulties were associated with enhanced negative affect to unpleasant events and a reduced response to neutral events. However, in individuals with unipolar depression, sleep difficulties were associated with increased negative affect for all everyday events (12).

It is possible that habitual ER strategy use is one mechanism linking mental health and sleep. Cognitive reappraisal, an antecedent-focussed ER strategy, involves the reappraisal of a potentially emotion-eliciting situation in a manner that changes its emotional impact. Expressive suppression is a response-focussed ER strategy that involves inhibiting ongoing emotion-expressive behavior (13). In laboratory paradigms investigating strategies aimed at modifying affect, cognitive reappraisal has been shown to be more effective than expressive suppression [see Ref. ( for a review]. ER has consistently been linked to affect in both clinical and non-clinical populations. Individuals who use maladaptive ER strategies including expressive suppression experience higher levels of internalizing symptoms including depressive and anxiety-related symptoms, whereas individuals who adopt adaptive ER strategies including cognitive reappraisal display fewer symptoms [e.g. Refs. (15–18)]. Further, cognitive reappraisal, an adaptive ER strategy, has been shown to offer protection against the development of psychopathologies, while the maladaptive ER strategy, expressive suppression, is linked to increased likelihood of developing an affective disorder [see (3) for a meta-analysis].

The neuroscientific study of ER has predominantly focussed on the investigation of the neural mechanisms underpinning cognitive reappraisal. At the neural level, in healthy individuals, ER has consistently been shown to recruit brain regions involved in top-down cognitive control (e.g. regions of the prefrontal cortex) as well as subcortical limbic structures including the amygdala [for reviews and meta-analyses see Ref. (19, 20)]). Recently, a meta-analysis of patients with mood or anxiety disorders demonstrated abnormal decreases and increases in the activation of brain networks involved in ER (21). Additionally, increased use of ER can predict stronger anatomical connections between the amygdala and PFC, while trait anxiety is negatively correlated with amygdala–PFC connectivity (22). Moreover, increased activation of the dorsolateral prefrontal cortex during ER can predict improvements in depressive symptoms in major depressive disorder (23). These findings at both the behavioral and neural level (including both functional and anatomical investigations) demonstrate that ER is linked to affect and psychopathology. Specifically, expressive suppression can act as a risk factor for the development of psychopathologies while cognitive reappraisal offers protection against the development of affective disorders.

Reduced focus has been devoted to the investigation of the links between specific ER strategies and sleep quality. Studies investigating links between expressive suppression, cognitive reappraisal and sleep quality have adopted self-report psychometric questionnaires and laboratory paradigms. Reduced sleep quality has been shown to be related to increased habitual use of expressive suppression, whereas sleep quality was not related to habitual use of cognitive reappraisal (24). However, converging evidence demonstrates that an evening chronotype, commonly associated with reduced sleep duration and quality, particularly in those working morning/regular day shifts (25–28), is associated with depression in the general population [e.g. Refs. (29, 30)]. Recently, an evening chronotype was associated with increased habitual use of expressive suppression, while a morning chronotype was associated with increased habitual use of cognitive reappraisal and reduced use of expressive suppression (31). These initial studies adopting psychometric approaches demonstrate links between habitual ER strategy use and sleep quality/circadian profiles. Like the research investigating the neural mechanisms underpinning ER, research adopting laboratory paradigms has focused on the ER strategy cognitive reappraisal, revealing mixed findings. Mauss et al. (32) suggested that poorer sleep quality is associated with lower cognitive reappraisal ability, whereas others have demonstrated that sleep quality scores are not related to cognitive reappraisal ability (33). Findings are also mixed in studies that have employed sleep restriction; sleep restriction has been associated with a decrease in self-reported cognitive reappraisal success (34), but did not impact adolescents’ ability to use cognitive reappraisal (35). Consequently, the exact relationships between ER and sleep quality are not yet fully understood.

The aim of the current study is to investigate the links between affect, ER, and sleep in healthy individuals. We predicted that within a healthy population, habitual use of cognitive reappraisal, a beneficial and protective ER strategy, would predict better sleep quality. In contrast, we hypothesized that habitual use of expressive suppression, a maladaptive ER strategy, would predict poor sleep quality. At present, however, it is not known whether affect can mediate the relationship between ER and sleep quality, and we therefore extend this approach to investigate whether positive and negative affect can mediate the impact of cognitive reappraisal and expressive suppression on sleep quality. Understanding the links between affect, ER, and sleep quality in healthy populations will help to guide future research on how these processes are altered in clinical populations.

## Materials and Methods

### Participants

An opportunity sample of 89 undergraduate students (59 female) aged 18–47 years (*M* = 22.50, *SD* = 4.54) from the University of Salford participated in this study. Gender and age information were omitted by 7 individuals. Participants read a Participant Information Sheet before providing informed consent *via* voluntary completion of the questionnaires. Ethical approval was obtained from the School of Health Sciences and School of Health & Society Ethics Committee at University of Salford. Where appropriate participants received course credit for completion of the questionnaires (n = 14).

### Design

The study adopted a cross-sectional correlational design with five variables; positive affect, negative affect, cognitive reappraisal, expression suppression, and sleep quality.

### Materials

Participants completed self-report questionnaires to record levels of positive and negative affect (Positive and Negative Affect Schedule; PANAS) (36), habitual use of ER strategies (Emotion Regulation Scale; ERQ) (13), and sleep quality (Pittsburgh Sleep Quality Index; PSQI) (37). The PANAS consists of 20 words used to describe positive and negative feelings and emotions. Participants are required to rate the extent to which a word describes how they feel on a 5-point Likert scale “indicate to what extent you feel this way right now, that is, at the present moment” ranging from 1 (very slightly) to 5 (extremely). The PANAS provides a measure of positive affect (10 items) and negative affect (10 items), with the responses summed for each measure providing a minimum score of 10 (indicative of lower affect) and a maximum score of 50 (indicative of higher affect). Watson et al. (36) report moderately good reliability and validity. Across 6 differing time frames, for both subscales, Cronbach’s alpha coefficient exceeded 0.84 in all instances. Test–retest reliability is 0.68 for positive affect and 0.71 for negative affect. The two subscales have been shown to be independent (*r* = −0.09).

The ERQ is a 10-item questionnaire that measures respondents’ tendency to regulate their emotions *via* two different ER strategies; cognitive reappraisal and expressive suppression (e.g. “When I’m faced with a stressful situation, I make myself think about it in a way that helps me stay calm”). Responses are provided on a 7-point Likert scale ranging from 1 (strongly disagree) to 7 (strongly agree). Six items relate to cognitive reappraisal providing a minimum score of 6 (indicating lower use of cognitive reappraisal) and a maximum score of 42 (indicating higher use of cognitive reappraisal). Four items relate to expressive suppression providing a minimum score of 4 (indicating lower use of expressive suppression) and a maximum score of 28 (indicating higher use of expressive suppression). Alpha reliabilities for the ERQ are 0.70 for cognitive reappraisal and 0.73 for expressive suppression. Test–retest reliability was 0.69 for both subscales.

The PSQI is a 19-item questionnaire that measures the patterns and quality of sleep. Questions include 4-point Likert scales for frequency or severity of problem and by reporting times (e.g. “how many hours of actual sleep did you get at night?”). Individuals are instructed that the questions (e.g. “during the past month, how often have you had trouble sleeping because you cannot get to sleep within 30 minutes?”) relate to “usual sleep habits during the past month only. Your answers should indicate the most accurate reply for the majority of days and nights in the past month”. The total score is calculated by summing seven subscale scores providing a minimum possible score of 0 and a maximum possible score of 21. The PQSI has a cutoff score where a total score of < 6 indicates good quality sleep and a score of > 5 indicates poor quality sleep. A Cronbach’s alpha for the PSQI components of 0.83 indicates a high level of internal homogeneity. Further, the PSQI has a diagnostic sensitivity of 89.6% and specificity of 86.5% (kappa = 0.75, *p* < 0.001) in distinguishing good and poor sleepers.

### Procedure

The questionnaires were uploaded onto Bristol Online Surveys, an online platform used to collect survey data. Participants viewed a Participant Information Sheet before completing the questionnaires. Alternatively, participants completed the questionnaires face-to-face by arranging an appointment with the researcher (n = 18). All participants had the opportunity to seek clarification before completing the questionnaires.

### Analysis

After checking the data met parametric assumptions (see *Results* section), relationships between variables were initially analysed using correlational analyses. Where significant relationships were evident, and regression model assumptions had been checked (see *Results* section), regression and mediation analyses were conducted. This approach allowed the investigation of whether expressive suppression and cognitive reappraisal were able to significantly predict sleep quality. The mediation analyses tested whether positive affect and/or negative affect were able to significantly mediate any relationships between habitual emotional regulation strategy use and sleep quality.

## Results

Descriptive statistics for the questionnaire measures are presented in **Table 1**. Kolmogorov-Smirnov test was used to assess normality suggesting that PSQI scores were not normally distributed, *D* (89) = 0.119, *p* = 0.003. Consequently, Spearman’s correlational analyses were conducted to quantify the relationships between emotional factors and sleep quality. PSQI score was positively correlated with expressive suppression, *r*_s_ (87) = 0.278, *p* = 0.004, demonstrating that as habitual expressive suppression use increases sleep quality decreases. The relationship between PSQI score and habitual cognitive reappraisal use approached significance, *r*_s_ (87) = −0.157, *p* = 0.071. Positive affect was negatively correlated with PSQI score, *r*_s_ (87) = −0.305, *p* = 0.002, demonstrating that greater levels of positive affect were related to improved sleep quality. Finally, negative affect was positively correlated with PSQI score, *r*_s_ (87) = 0.509, *p* < 0.001, suggesting that lower levels of negative affect were related to improved sleep quality.

**Table 1:** Descriptive statistics for emotional factors and sleep quality.

Pittsburgh Sleep Quality Index	8.0 ± 4.1
Emotion Regulation Scale Cognitive Reappraisal subscale	27.0 ± 7.3
Emotion Regulation Scale Expressive Suppression subscale	15.3 ± 5.1
Positive and Negative Affect Schedule Positive Affect subscale	26.7 ± 8.3
Positive and Negative Affect Schedule Negative Affect subscale	19.1 ± 9.0

Values are means ± standard deviations.

**Figure 1:**
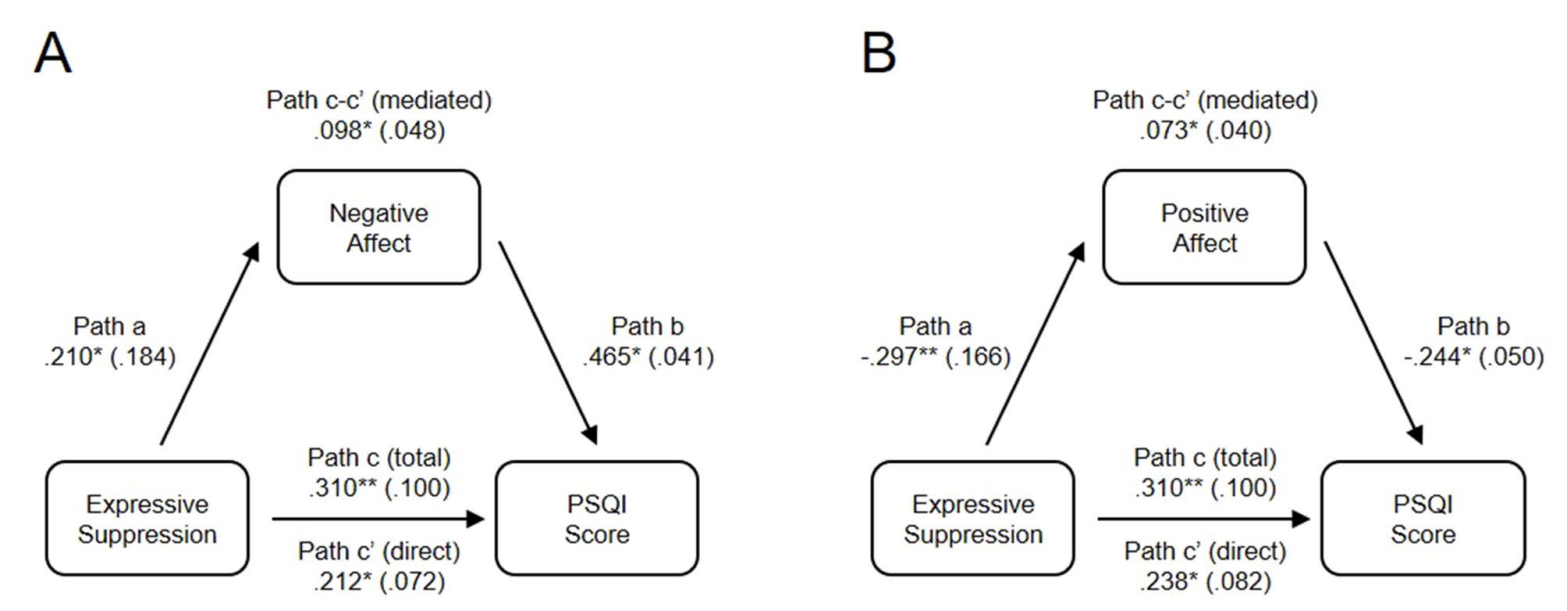
Partial mediation between expressive suppression and sleep quality by both **(A)** negative affect, and **(B)** positive affect. Data values are regression betas for specific pathways, with standard errors shown in parentheses. High PSQI scores indicate poor sleep. *p < .05, **p < .01.

As significant correlations were present between expressive suppression, positive affect, negative affect, and sleep quality, regression analyses were conducted to test the hypotheses that affect mediates the relationship between expressive suppression and sleep quality (**Table 2** and **Figure 1**). Data met the assumptions relevant for regression analysis. An analysis of standardized residuals demonstrated that the data contained no outliers; standardized residual minimum = −1.903, standardized residual maximum = 2.517. Collinearity was not a concern as all tolerance scores were > 0.878, and all variance inflation factors < 1.139. The data also met the assumption of independent errors as indicated by a Durban-Watson value of 1.675. The histogram of standardized residuals indicated that the data contained approximately normally distributed errors, as did the normal P–P plot of standardized residuals, which showed points were close to the line. The scatterplot of standardized residuals showed that the data met the assumptions of homogeneity of variance and linearity. Finally, the data also met the assumption of non-zero variances with all scores > 16.511.

**Table 2:** Positive and negative affect as mediators between expressive suppression and sleep quality.

Path name	Association	Mediator: PANAS-NA	Mediator: PANAS-PA
Path a	ERQ-ES → mediator	0.210*	−0.297**
Path b	Mediator → PSQI	0.465*	−0.244*
Path c	ERQ-ES → PSQI (total)	0.310**	0.310**
Path c’	ERQ-ES → PSQI (direct)	0.212*	0.238*
Path c-c’	ERQ-ES → PSQI (mediated)	0.098*	0.073*

PSQI, Pittsburgh Sleep Quality Index; ERQ-ES, Emotion Regulation Scales Expressive Suppression subscale; PANAS-PA, Positive and Negative Affect Schedule Positive Affect subscale and PANAS-NA, Positive and Negative Affect Schedule Negative Affect subscale. *p < .05, **p < .01.

In model 1, standardized regression betas indicated that expressive suppression was a significant predictor of negative affect, β = 0.210, *SE* = 0.184, *p* = 0.043 (**Figure 1A**, path a), and that negative affect significantly predicted PSQI score, β = .465, *SE* = 0.041, *p* < 0.001 (**Figure 1A**, path b). Irrespective of affect, the total effect of expressive suppression on sleep quality was significant, β = 0.310, *SE* = 0.100, *p* = 0.004 (**Figure 1A**, path c). When controlling for negative affect, expressive suppression still predicted PSQI score, β = .212, *SE* = 0.072, *p* = 0.020 (**Figure 1A**, path c’), consistent with partial mediation. Approximately 30% of the variance in sleep quality was accounted for by the predictors (R^2^ = 0.303). The indirect mediated path (**Figure 1A**, path c–c’) was tested using a bootstrap estimation approach with 1,000 samples. This result indicated that the indirect coefficient was significant, β = −0.098, *SE* = 0.048, *p* = 0.018, 95% CI = −0.184, −0.028, confirming mediation. Together, these analyses reveal that negative affect partially mediates the effect of expressive suppression on sleep quality (**Table 2**).

Model 2 indicated that expressive suppression was a significant predictor of positive affect, β = −0.297, *SE* = 0.166, *p* = 0.004, (**Figure 1B** path a), and that positive affect significantly predicted PSQI score, β = −0.244, *SE* = 0.050, *p* = 0.018, (**Figure 1B**, path b). Irrespective of affect, the total effect of expressive suppression on sleep quality was significant, β = 0.310, *SE* = 0.100, *p* = 0.004 (**Figure 1A**, path c). When controlling for positive affect, expressive suppression still predicted PSQI score, β = .238, *SE* = 0.082, *p* = 0.021 (**Figure 1B**, path c’), consistent with partial mediation. Approximately 15% of the variance in sleep quality was accounted for by the predictors (R^2^ = 0.151). The indirect mediated path (**Figure 1B**, path c–c’) was tested using a bootstrap estimation approach with 1,000 samples, demonstrating that the indirect coefficient was significant, β = 0.073, *SE* = 0.040, *p* = 0.010, 95% CI = −0.162, −0.024, confirming mediation. Together, these analyses reveal that positive affect partially mediates the effect of expressive suppression on sleep quality (**Figure 1**).

## Discussion

To our knowledge the current study is the first to investigate if affect mediates the relationship between habitual ER use and sleep quality in a healthy population. Correlational analyses demonstrated that increased positive affect was associated with improved sleep quality, whereas greater negative affect scores were associated with reduced sleep quality. Increased use of expressive suppression was also associated with poorer sleep quality, while the relationship between cognitive reappraisal and sleep quality was non-significant. Regression and mediation analyses revealed that increased habitual use of expressive suppression, theorized to be a maladaptive ER strategy, was shown to predict poorer sleep quality. Finally, both positive and negative affect were found to mediate the effects of expressive suppression on sleep quality.

The finding that expressive suppression predicts poorer sleep quality is consistent with recent work in an adolescent sample (38) and adult population (24), but here we extend this using mediation analysis to report the novel finding that both positive and negative affect influence the effect of expressive suppression on sleep quality. Moreover, we demonstrate that negative affect explains more of the variance in sleep quality than positive affect. Previously, using structural equation modelling, it has been shown that while negative affect was able to predict sleep quality, positive affect was not (39). Here, however, we do report a significant influence of positive affect on sleep quality, but consistent with this previous work, negative affect had a more prominent influence. This observation suggests that therapies targeting the regulation of negative affect may be more successful in improving sleep quality compared to interventions aimed at improving positive affect. Further research will be necessary to elucidate the specific negative affect factors responsible for this relationship. For example, negative affect is associated with the experience of anxiety, fear, sadness, depression, and stress (40, 41). While general negative affect is strongly linked to affective disorders, it often has differential relationships with specific psychopathologies (42). Thus, it will be important to investigate which specific secondary level negative affect factors (e.g. anxiety, fear, sadness, depression, or stress) influence the relationship between ER strategy use and sleep quality. Such research may help to develop more efficacious interventions. The observation that cognitive reappraisal is not significantly correlated with sleep quality or affect is surprising given that this ER strategy has been shown to offer protection against the development of psychopathologies. Nonetheless, this observation is supported by recent work suggesting that cognitive reappraisal is not correlated with sleep quality (24). However, it should be noted that the correlation between cognitive reappraisal and sleep quality approached significance (*p* = 0.071), and further studies are needed to explore the relationships between cognitive reappraisal and sleep quality in healthy and clinical populations.

Sleep disturbances and psychopathologies are often comorbid. It has been suggested both that treatments aimed at reducing negative affect may improve the effectiveness of interventions for insomnia, and that the efficacy of interventions aimed at preventing or treating affective disorders may be enhanced by simultaneously addressing sleep quality (39). The current findings in healthy individuals support this claim. Recently, emotion regulation therapy has shown reductions in symptom severity for generalized anxiety disorder and major depressive disorder patients (43). Moreover, functional changes in brain regions located within the salience network and default mode network, both of which show disruptions in depression and anxiety patients, have shown treatment-related changes following emotion regulation therapy corresponding with clinical improvements (44). Our current research indicates that future studies evaluating the potential benefits of emotion regulation therapy should simultaneously assess improvements in sleep quality and changes in psychopathology.

The current research presents novel findings and furthers our understanding of the relationships between ER, affect, and sleep quality. However, a few limitations warrant consideration. The study adopted a cross-sectional correlational design preventing causal inferences regarding the nature of the relationships between ER, affect, and sleep quality. Consequently, experimental, longitudinal and intervention studies are required to provide such causal inferences. Additionally, the study employed a convenience sample of healthy individuals, and so caution must be taken when applying these findings to clinical populations. Nonetheless, the findings reported here provide a basis for similar research within clinical groups. The study did not employ any inclusion or exclusion criteria, such as screening for psychological conditions or sleep disorders, and so it is possible that other variables could mediate the relationships between ER and sleep quality. Demographic variables such as age and gender were not identified on an *a priori* basis as variables of interest and so were omitted from the analytical framework. Sleep quality has been shown to reduce with age [e.g. Ref. (45)], while age- and gender-related differences in ER strategies are evident [e.g. Ref. (46–48)]. Studies including these demographic variables on an *a priori* basis are needed to investigate if age and gender influence the relationships between ER, affect and sleep quality.

Additionally, while the current study used established psychometric measures for assessing ER, affect and sleep quality, objective measures were not included. For instance, actigraphy, an objective measure of sleep, is not always correlated with self-report measures of sleep quality provided by the PSQI (49). Further, negative affect has been shown to be related to self-reported sleep but not objective measures of sleep (50). In the current study habitual ER strategy use was assessed *via* self-reports. Future studies can seek to assess ER using laboratory paradigms. Moreover, recently it has been argued that ER paradigms should also investigate the decision to deploy ER strategies, rather than using paradigms that only investigate instructed ER (51, 52). Adopting such approaches permits the investigation of individual differences in the decisions to use ER and will aid development of our knowledge of traits that can be targeted for clinical intervention.

The novel findings reported here in a healthy population provide the basis for progression to similar work in clinical populations with diagnosed sleep disturbances and/or affective disorders. Future research, utilizing clinical populations, will develop our understanding of the relationships between ER, affect and sleep quality and raise the possibility of potential new therapeutic tools for the treatment of both psychopathologies and sleep disorders.

Adopting mediation analyses, positive and negative affect were found to partially mediate the relationship between expressive suppression and sleep quality. These data suggest that including elements aimed at modulating affect, and in particular reducing negative affect, as well as minimizing habitual use of expressive suppression may improve the effectiveness of psychological interventions for patients with sleep disorders.

## Data Availability

The raw data supporting the conclusions of this manuscript will be made available by the authors, without undue reservation, to any qualified researcher.

## Ethics Statement

This study was carried out in accordance with the recommendations of School of Health Sciences and School of Health & Society Ethics Committee at University of Salford. Informed consent was inferred *via* voluntary completion of the questionnaires. The protocol was approved by the School of Health Sciences and School of Health & Society Ethics Committee at University of Salford.

## Author Contributions

IL: data collection. IL and RB study design. IL, RB and AH statistical analysis, manuscript preparation, interpretation of results. All authors agreed final manuscript submission.

## Conflict of Interest Statement

The authors declare that the research was conducted in the absence of any commercial or financial relationships that could be construed as a potential conflict of interest.
